# 30-year experience of Fontan surgery: single-centre’s data

**DOI:** 10.1186/s13019-017-0634-0

**Published:** 2017-08-09

**Authors:** Laurynas Bezuska, Virgilijus Lebetkevicius, Rita Sudikiene, Daina Liekiene, Virgilijus Tarutis

**Affiliations:** 1 Department of Cardiovascular Medicine, Vilnius University, Santariskiu 2, 08661 Vilnius, Lithuania; 2 Centre of Cardiac Surgery, Vilnius University Hospital Santariskiu Klinikos, Santariskiu 2, 08661 Vilnius, Lithuania

**Keywords:** Cavopulmonary connection, Gore-Tex® conduit, Single ventricle

## Abstract

**Background:**

The Fontan procedure has been modified several times since it was introduced into practice in 1968. As many patients now survive to adulthood, attention is directed towards their clinical status and late morbidity. We report our surgical experience of 30 years in Fontan procedures.

**Methods:**

From January 1985 to January 2015, 80 patients underwent Fontan surgery. Twenty-one patients received an atrio-pulmonary Fontan (Group I), four patients underwent total cavopulmonary connection (TCPC) with an intra-atrial lateral tunnel (Group II), six patients received extra-cardiac TCPC with an aortic homograft (group III) and 49 patients received extra-cardiac TCPC with an expanded polytetrafluoroethylene conduit. They were followed for early and late mortality, long-term survival, postoperative morbidity and reoperations.

**Results:**

The mean follow-up time was 7.4 ± 6.6 years. The Kaplan–Meier estimated 15-year survival rate was 42% in Group I, 50% in Group II, 83% in Group III and 94% in Group IV. The median length of stay in intensive care unit, intubation and chest drain stay time were 90 h (IQR, 46–119), 8 h (IQR, 6–16) and 18 days (IQR, 12–28) respectively. Early complications were bleeding (6), taken down of Fontan circulation (3) and acute heart failure managed by left heart bypass (1). Late-occurring morbidities included arrhythmias (6), protein-losing enteropathy (2), thromboembolism (2) and tracheal stenosis (1). Fourteen patients (18%) had redo Fontan procedures.

**Conclusion:**

Our series showed improving results after Fontan completion with excellent mid-term outcome after extra-cardiac TCPC with expanded polytetrafluoroethylene conduit. The long-term result should be followed.

## Background

More than 45 years have passed since Francis Fontan successfully performed an operation with the intention of redirecting systemic venous return into the pulmonary arteries without a pulmonary ventricle [1]. Nowadays this type of surgery is known as a Fontan procedure, there as circulation with a single ventricle is known as a Fontan circulation. However, Fontan cautioned in his paper the procedure was not an anatomical correction but a physiological pulmonary blood flow restoration [1]. Kreutzer modified the procedure, which is known as an atriopulmonary Fontan [2]. De Leval proved total cavopulmonary connection (TCPC) with a lateral tunnel to be a better option to atriopulmonary connection [3]. Marcelleti [4] and Corno [5] introduced modified Fontan with an extracardiac conduit, which is the mainstream surgery at present.

Despite favourable outcome of Fontan circulation, there are important circulatory limitations. Elevated central venous pressure and impaired cardiac output are consequences of this palliative circulation. This evokes progressive decline of likely all organ systems [6]. Specific long-term complications are described as plastic bronchitis, protein losing enteropathy [7], arrhythmias [8], liver fibrosis [9], thromboembolism [10], lymphatic insufficiency [11], altered bone density, decreased muscle mass [12], Fontan circulatory failure [13] and others. Goldberg pointed out the need of a multidisciplinary team of specialists for the long-term follow-up these unique patients [14].

We want to share our experience of Fontan procedures performed at our institution over a 30-year period. By our knowledge, this is the first report of such time scale in the population of the Baltic States.

## Methods

Children with functional single ventricle who underwent Fontan procedure from January 1985 to January 2015 were identified. All patients were operated at a single institution at Vilnius University Hospital Santariskiu Klinikos Heart Surgery Center, Lithuania. The cross-sectional retrospective study was reviewed and approved by the Vilnius Regional Biomedical Research Ethics Committee. Data was obtained by review of paper and electronic medical records from admission until the last follow-up.

Eighty patients (37 females and 43 males) were operated between January 1985 and January 2015. Median age at the first Fontan procedure was 4.6 years (interquartile range (IQR): 3.1–7.9). The youngest patient was 14 months and the oldest was 26.5 years of age. All patients were divided into 4 groups according to the type of the Fontan procedure. Twenty-one patients underwent atrio-pulmonary Fontan (Group I) between 1985 and 1998. Four children had an intra-atrial lateral tunnel (Group II) in 1993–2000. Six patients received extracardiac TCPC with an aortic homograft (Group III) in 1996–2005. The remaining 49 children (Group IV) were operated between 2000 and 2015 and received extra-cardiac TCPC with expanded polytetrafluoroethylene (PTFE) conduit (Table 1). Congenital diagnoses and their association between groups are listed in Table 2.

**Table 1:** Mortality rate in different types of Fontan procedures

Procedure	Group	Number of patients	Mortality, n		
			Early	Late	Total
Atrio-pulmonary Fontan	I	21	5 (24%)	5 (31%)	10 (48%)
TCPC with lateral tunnel	II	4	1 (25%)	1 (33%)	2 (50%)
TCPC with extra-cardiac Ao homograft	III	6	0 (0%)	1 (17%)	1 (17%)
TCPC with extra-cardiac expanded PTFE conduit	IV	49	2 (4%)	1 (2%)	3 (6%)
TOTAL	-	80	7 (9%)	9 (12%)	16 (20%)

*TCPC* total cavopulmonary connection, *PTFE* polytetrafluoroethylene

**Table 2:** List of diagnoses and associations

Diagnosis	Group I, *n* = 21	Group II, *n* = 4	Group III, *n* = 6	Group IV, *n* = 49
Type of morphological ventricle (LV/RV)	20/21	3/1	6/0	39/10
Tricuspid valve atresia	4	1	4	18
Single ventricle	14	1	-	10
Hypoplastic left heart syndrome	-	1	-	8
Pulmonary artery atresia	2	1	-	4
Other	1	-	2	9

*LV* left ventricle, *RV* right ventricle

### Definitions

Operative mortality was defined as death occurring within 30 days of surgery or before hospital discharge. Overall mortality was counted as early mortality combined with late mortality at follow up.

### Data analysis

The statistical software SPSS 21.0 for Windows (SPSS Inc. Chicago, Illinois, USA) was employed. Data is presented as medians and interquartile ranges due to the non-normal distribution of variables. Kaplan-Meier method was used to evaluate survival functions. A value of *p* < 0.05 was considered to be significant. The statistical analysis was not used between groups due to the different type of Fontan surgery, era of performed operations and inadequate and uneven number of patients in the groups.

## Results

The mean follow-up time was 7.4 ± 6.6 years. The longest follow up time for one of the patients was 25.8 years. Median age was 4.6 (IQR, 3.1–7.9) years. The youngest patient undergoing Fontan procedure was 14 months old (who received extra-cardiac TCPC with 20 mm expanded PTFE conduit), while the oldest (26 years old) underwent atrio-pulmonary Fontan with fenestration. Six patients in Group I and two patients in Group IV were lost at follow-up. Seventeen patients (35%, 17/49) had their extra-cardiac TCPC younger than 3 years of age. Median weight was 14.7 kg (IQR, 12.2–18). Median preoperative mean pulmonary artery pressure was 13 mmHg (IQR, 11–15) and pulmonary vascular resistance – 1 WU*m^2^ (IQR, 1–1.4). Fenestration was created in 40 (50%) patients. The median length of stay in intensive care unit, intubation and chest drain stay time were 90 h (IQR, 46–119), 8 h (IQR, 6–16) and 18 days (IQR, 12–28) respectively. Nine patients from the early era underwent Fontan procedure as a primary operation. All others had initial palliation procedures before Fontan surgery. Surgical procedures at the initial palliation are listed in Table 3. One patient gave birth to a healthy baby by Caesarean section 22 years later after initial atrio-pulmonary Fontan procedure. Warfarin was routinely used for 6 months postoperatively in all our patients.

**Table 3:** Staged procedures at the initial palliation

Procedure	Group I, *n* = 21	Group II, *n* = 4	Group III, *n* = 6	Group IV, *n* = 49
Modified Norwood	-	-	-	8
Modified Blalock–Taussig shunt	11	1	3	7
Central shunt	1	1	-	9
Pulmonary artery banding	1	1	2	14
Initial Glenn	-	1	-	11
Fontan procedure	8	-	1	-

### Morbidity

The complete list of complications is shown in Table 4. Early complications were bleeding (6), taken down of Fontan circulation (3), chylothorax (1), paresis of hemidiaphragm (1), focal epilepsy (1) and acute heart failure managed by left heart bypass (1). Late-occurring morbidity included arrhythmias (6), protein-losing enteropathy (2), thromboembolism (2), aortic insufficiency managed by valvuloplasty (1) and tracheal stenosis (1).

**Table 4:** List of complications after Fontan procedures

Complication	Number
Taken down of Fontan	3
Redo Fontan	15
Protein-losing enteropathy	2
Bleeding complications	6
Trombembolism	2
Heart failure managed by left heart bypass	1
Aortic insufficiency managed by valvuloplasty	1
Focal epilepsy	1
Tracheal stenosis	1
Chylothorax	1
Arrhythmias	6
Paresis of hemidiaphragm	1

These complications are not mutually exclusive; some patients had few complications

Fourteen patients (18%, 14/80) had redo Fontan procedures with one early mortality (7%, 1/14). The majority of the patients (64%, 9/14) were from an atrio-pulmonary Fontan group. One child had a redo Fontan twice. Three patients in Group III required redo procedures as an aortic homograft became calcified and stenotic. Three children from Group IV had redo TCPC to upgrade to a larger 22 mm diameter conduit as extracardiac expanded PTFE conduits had become obstructed. The distribution of redo Fontan procedures between groups is shown in Table 5.

**Table 5:** Distribution of Redo Fontan procedures

Redo procedure	TCPC with ePTFE tube, n	TCPC, lateral tunnel, n	Atrio-pulmonary Fontan
Procedure			
Atrio-pulmonary Fontan, *n* = 21	4	4	1
TCPC with lateral tunnel, *n* = 4	0	0	0
TCPC with extra-cardiac aortic homograft, *n* = 6	3	0	0
TCPC with extra-cardiac expanded PTFE conduit, *n* = 49	3	0	0

*TCPC* total cavopulmonary connection, *PTFE* polytetrafluoroethylene. One patient had a redo Fontan procedure twice

### Mortality

**Fig. 1:**
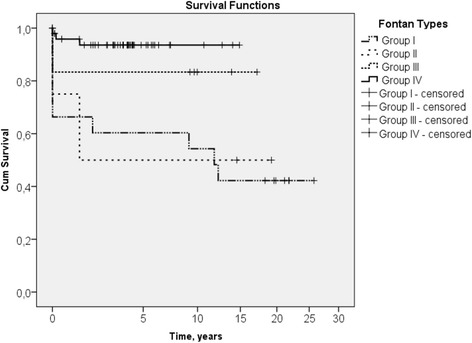
Kaplan-Meier survival in different types of Fontan procedures. Plot adjusted by power. Group I – Atrio-pulmonary Fontan. Group II – Lateral Tunnel. Group III – Total cavopulmonary connection (TCPC) with aortic homograft. Group IV – TCPC with expanded PTFE conduit

Seven (7/80, 9%) patients died in the early postoperative period, while nine (9/73, 12%) died in the late postoperative period. The majority of dead patients (10 out of 16) were from early era – Group I (Table 1). The Kaplan Mayer survival plot shows dramatically improving mortality rates between different groups and with cumulative survival of 94% in Group IV (Fig. 1). Five early mortality cases in Group I were associated with heart insufficiency and multiple organ failure. Five patients died late in Group I. One child died 11 years later after Fontan procedure secondary to right atrial thrombosis. Another child died 12 years later due to unintentional drowning while swimming in a lake. A third patient died 1.5 years after initial failed Fontan that needed to be converted to Glenn. This patient died in early postoperative period after redo Fontan due to heart failure. The other two patients died late due to unconfirmed reasons. There was one early death due to multiple organ failure and one late death due to cardiac arrhythmia in Group II. The only one patient died late in Group III for unknown reason. Three children out of 49 died in Group IV. One early death occurred on the second postoperative day due to sudden cardiac arrest and unsuccessful resuscitation. The second early mortality case appeared 6 weeks post-Fontan procedure because of progressing heart failure and severe draining. The predisposition of the late death was severe protein losing enteropathy and the child died due to acute heart and multiple organ failure two days later after Redo TCPC.

## Discussion

The outcome of our patients improved significantly with time. Our data suggests extracardiac TCPC can be performed with low risk and provides excellent survival in the early and mid-term. Our 94% survival rate corresponds with recently published 92–98% mid-term survival reports [15–17]. Although patients need to be followed closely for long-term outcomes. Elevated central venous pressure and impaired cardiac function eventually affect all Fontan survivors [18]. A multidisciplinary team approach to follow these patients from early postoperative period is highly recommended [14, 19].

The optimal age for Fontan procedure is dubious nowadays. Some contemporary centres aim to complete Fontan surgery in early childhood [20–22]. Our main hemodynamic criteria for Fontan completion were pulmonary artery pressure lower than 15 mmHg, pulmonary vascular resistance less than 4 WU*m2 and presence of aortopulmonary collaterals. The presence of collaterals was one of the trigger for early Fontan operation. Other factors, which determined our choice for earlier Fontan, were clinical symptoms as increasing cyanosis, decreasing physical activity or failure to thrive. In our group, 17 patients underwent extra-cardiac TCPC younger than 3 years of age. No significant difference was noticed in mortality and morbidity compared the outcome with the older patients [23]. This data corresponds with other authors’ results. They also stated that early TCPC could improve exercise capacity and hemodynamics [20, 24].

Our study included four types of Fontan procedures with relatively small numbers of the patients in the first three groups. Statistical calculations were not available for risk factors in different groups because of the small number of the participants. Additional obstacle for risk analysis was the fact that surgeries were performed in different eras and long period by different surgeons. The aim of our study was to share our overall experience in Fontan surgeries.

The most frequent early and late complications were bleeding, arrhythmias, protein-losing enteropathy and thromboembolism in our study. Type and incidence of morbidity corresponded to other authors results [10, 17, 20]. Our early morbidity occurred in the first 30 days post procedure or during the same admission. We recorded few late complications which occurred in different times post-surgical intervention and we could not find any correlations. One of the reasons of small amount of late complications is relatively short mean follow-up time (7.4 ± 6.6 years). Our study did not show any correlation of the fenestration with the late complications. Recent studies underline the importance of hepatic complications in Fontan circulation [9, 25, 26]. We did not confirm any significant liver damage in our group but it could be due to insufficient evaluation of hepatic fibrosis and its subclinical manifestation [18]. Magnetic resonance elastography is a promising non-invasive diagnostic tool to evaluate hepatic fibrosis and could be used in future practice to help evaluate liver damage following Fontan patients [26]. Our standard protocol included regular follow up visits at cardiology clinic with cardiac echography and blood tests. Decreasing protein level, increasing hypoxia and worsening common valve regurgitation or stenosis were triggers for more detailed examination which could include heart MRI, CT or cardiac catheterization. The choice for these investigations depended on individual patient status.

The majority of our redo Fontan procedures (9 of 14) were from an atrio-pulmonary Fontan group. These patients needed additional procedures due to enlarged right atrium and occurring arrhythmias. Eight patients have had a Fontan conversion procedure. Four patients were converted from atriopulmonary Fontan to lateral tunnel Fontan and the rest four patients were converted from atriopulmonary Fontan to extracardiac Fontan with ePTFE conduit. Three patients from the extracardiac TCPC group with expanded PTFE conduit needed an upgrade to a bigger conduit (size 22). These children were operated at early stage after introduction of the expanded PTFE conduit in our hospital. The later patients received bigger conduits (at least size 18) and none of them needed reoperation due to conduit stenosis. Our results revealed that conversion of a failing Fontan connection to a TCPC could be accomplished safely and successfully. Park [27] presented similar results recently.

Failing Fontan circulation at long term is one of the biggest challenges. Heart transplantation is one of the options. Although, in our group none of the patients underwent heart transplantation after Fontan procedure. In the recent study, Kanter did not identify any early or mid-term disadvantage for children undergoing heart transplantation after a previous Fontan procedure, despite more complex transplant operations [28]. The author stated that carefully selected children with a failing Fontan circulation could do as well as other children with heart transplantation. While Jaquiss proposed a right-sided sub-pulmonary ventricular assist device which could be a four stage management or destination therapy in the Fontan patients [29].

The adult population with Fontan circulation is increasing with time due to better survival [30]. Schilling predicts population of patients alive after a Fontan procedure will double over the next 20 years [31]. This increases demand of medical and non-medical care for these patients. One should prepare for the bigger demand of care particularly in adult congenital and heart failure centres.

### Study limitations

A retrospective nature and relatively small number of patients are limiting factors for this single institutional study. The more contemporary patients have limited follow-up so late morbidity and mortality may be underrepresented. The change of general improvement in surgical technique and perioperative care has occurred with time that may alter the long-term results.

## Conclusion

Mid-term outcomes after Fontan completion are good and comparable in our centre. Patients need to be followed up for long term. The multidisciplinary approach is of the most benefit. The increasing quantity of adult Fontan patients creates challenges for adult congenital follow up team.

## Abbreviations

IQR: Interquartile range
LV: Left ventricle
PTFE: Polytetrafluoroethylene
RV: Right ventricle
TCPC: Total cavopulmonary connection
